# Crystal structure of methyl (*E*)-2-(1-methyl-2-oxoindolin-3-yl­idene)acetate

**DOI:** 10.1107/S2056989015003217

**Published:** 2015-02-21

**Authors:** M. P. Savithri, P. S. Yuvaraj, B. S. R. Reddy, R. Raja, A. SubbiahPandi

**Affiliations:** a Department of Physics, Queen Mary’s College (Autonomous), Chennai 600 004, India; b University of Madras, Industrial Chemistry Laboratory, Central Leather Research Institute, Adyar, Chennai 600 020, India; c Department of Physics, Presidency College (Autonomous), Chennai 600 005, India

**Keywords:** crystal structure, indole, 3-substituted indoles, C—H⋯O hydrogen bonds, C—H⋯π inter­actions, π–π stacking inter­actions

## Abstract

The title compound, C_12_H_11_NO_3_, is essentially planar, with the mean plane of the acetate side chain [–C—C(=O)—O—C] being inclined to the mean plane of the indole ring system by 12.49 (7)°. The five- and six-membered rings of the indole group are almost coplanar, making a dihedral angle of 1.76 (8)°. The conformation about the C=C bond is *E* and there is an intra­molecular C—H⋯O hydrogen bond present. In the crystal, mol­ecules are linked by pairs of C—H⋯O hydrogen bonds forming inversion dimers, with an *R*_2_^2^(16) ring motif. The dimers are linked by a second pair of C—H⋯O hydrogen bonds, enclosing *R*_2_^2^(16) ring motifs, forming ribbons lying parallel to (-114). The ribbons are linked *via* C—H⋯π inter­actions, forming a three-dimensional structure.

## Related literature   

For general background to the synthesis of 3-substituted indole derivatives as precursors of potent anti-inflammatory and analgesic agents, see: Radwan *et al.* (2007 ▸). For related structures, see: Bhella *et al.* (2009 ▸); Hou & Li (2011 ▸).
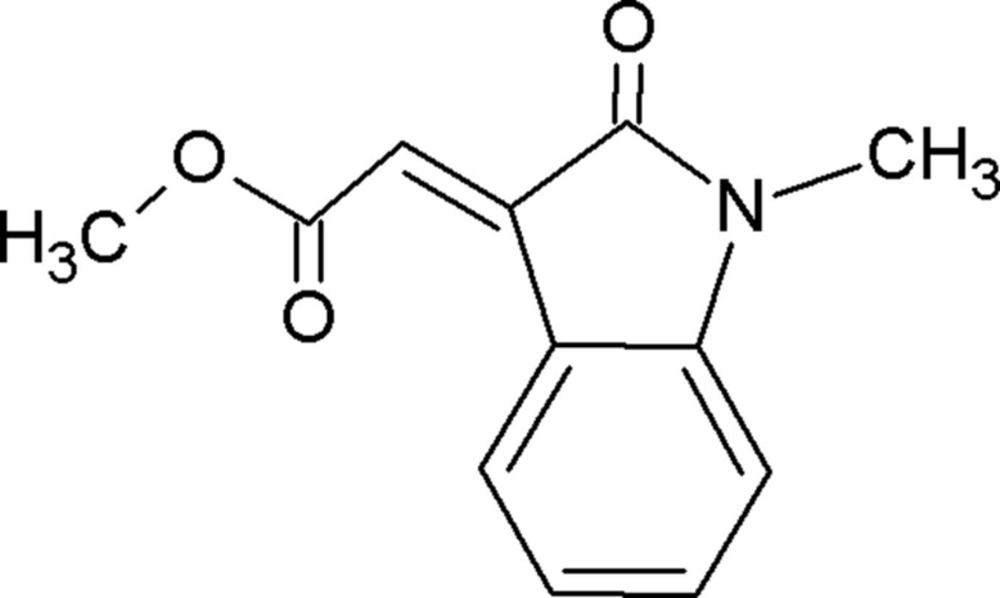


## Experimental   

### Crystal data  

C_12_H_11_NO_3_
*M* *_r_* = 217.22
Monoclinic, 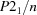

*a* = 11.6814 (7) Å
*b* = 5.6106 (4) Å
*c* = 16.5299 (11) Å
β = 108.713 (2)°
*V* = 1026.09 (12) Å^3^
*Z* = 4
Mo *K*α radiation
μ = 0.10 mm^−1^
*T* = 293 K
0.35 × 0.30 × 0.30 mm

### Data collection  

Bruker Kappa APEXII CCD diffractometer
Absorption correction: multi-scan (*SADABS*; Bruker, 2004 ▸) *T*_min_ = 0.948, *T*_max_ = 0.955
14793 measured reflections
1809 independent reflections
1528 reflections with *I* > 2σ(*I*)
*R*_int_ = 0.023

### Refinement  

*R*[*F* ^2^ > 2σ(*F* ^2^)] = 0.033
*wR*(*F* ^2^) = 0.093
*S* = 1.05
1809 reflections
148 parameters
H-atom parameters constrained
Δρ_max_ = 0.18 e Å^−3^
Δρ_min_ = −0.13 e Å^−3^

### 

Data collection: *APEX2* (Bruker, 2004 ▸); cell refinement: *APEX2* and *SAINT* (Bruker, 2004 ▸); data reduction: *SAINT* and *XPREP* (Bruker, 2004 ▸); program(s) used to solve structure: *SHELXS97* (Sheldrick, 2008 ▸); program(s) used to refine structure: *SHELXL2014* (Sheldrick, 2015 ▸); molecular graphics: *ORTEP-3 for Windows* (Farrugia, 2012 ▸), *PLATON* (Spek, 2009 ▸) and *Mercury* (Macrae *et al.*, 2008 ▸); software used to prepare material for publication: *SHELXL2014* and *PLATON*.

## Supplementary Material

Crystal structure: contains datablock(s) global, I. DOI: 10.1107/S2056989015003217/su5085sup1.cif

Structure factors: contains datablock(s) I. DOI: 10.1107/S2056989015003217/su5085Isup2.hkl

Supporting information file. DOI: 10.1107/S2056989015003217/su5085Isup3.cml

**Fig. 1.**
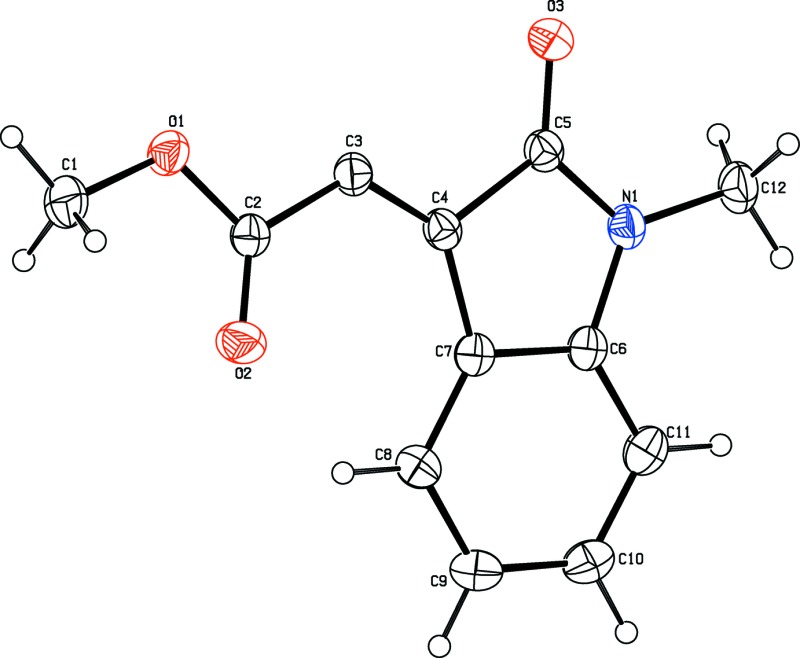
The molecular structure of the title compound, with atom labelling. Displacement ellipsoids are drawn at the 30% probability level.

**Fig. 2.**
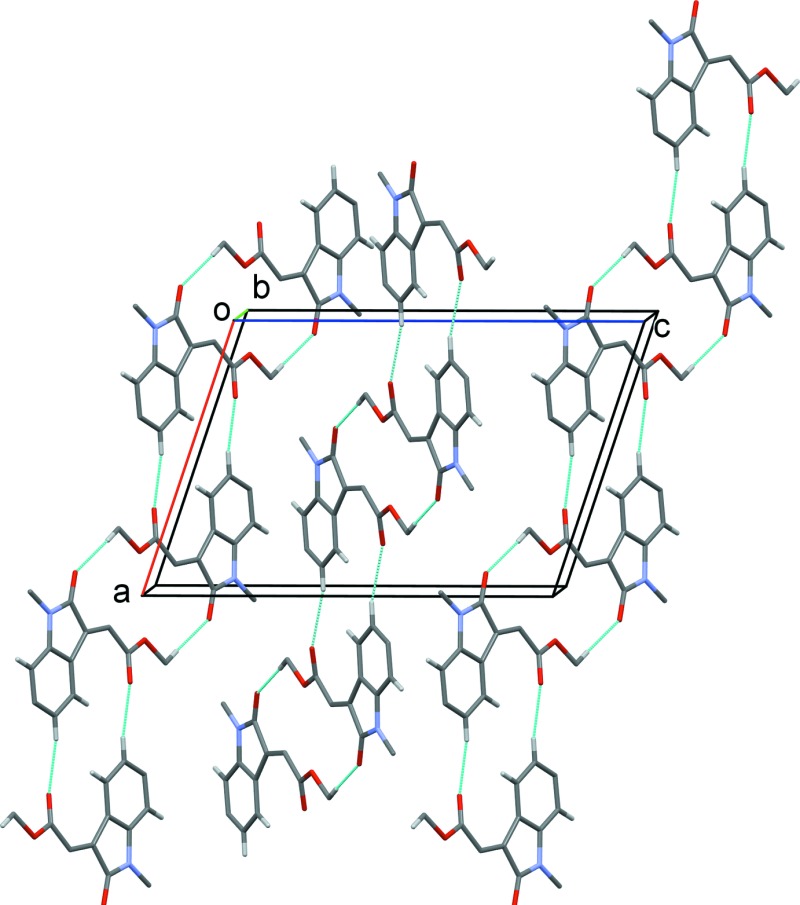
A partial view along the b axis of the crystal packing of the title compound. The hydrogen bonds are shown as dashed lines (see Table 1 for details; H atoms not involved in hydrogen bonding have been omitted for clarity).

**Fig. 3.**
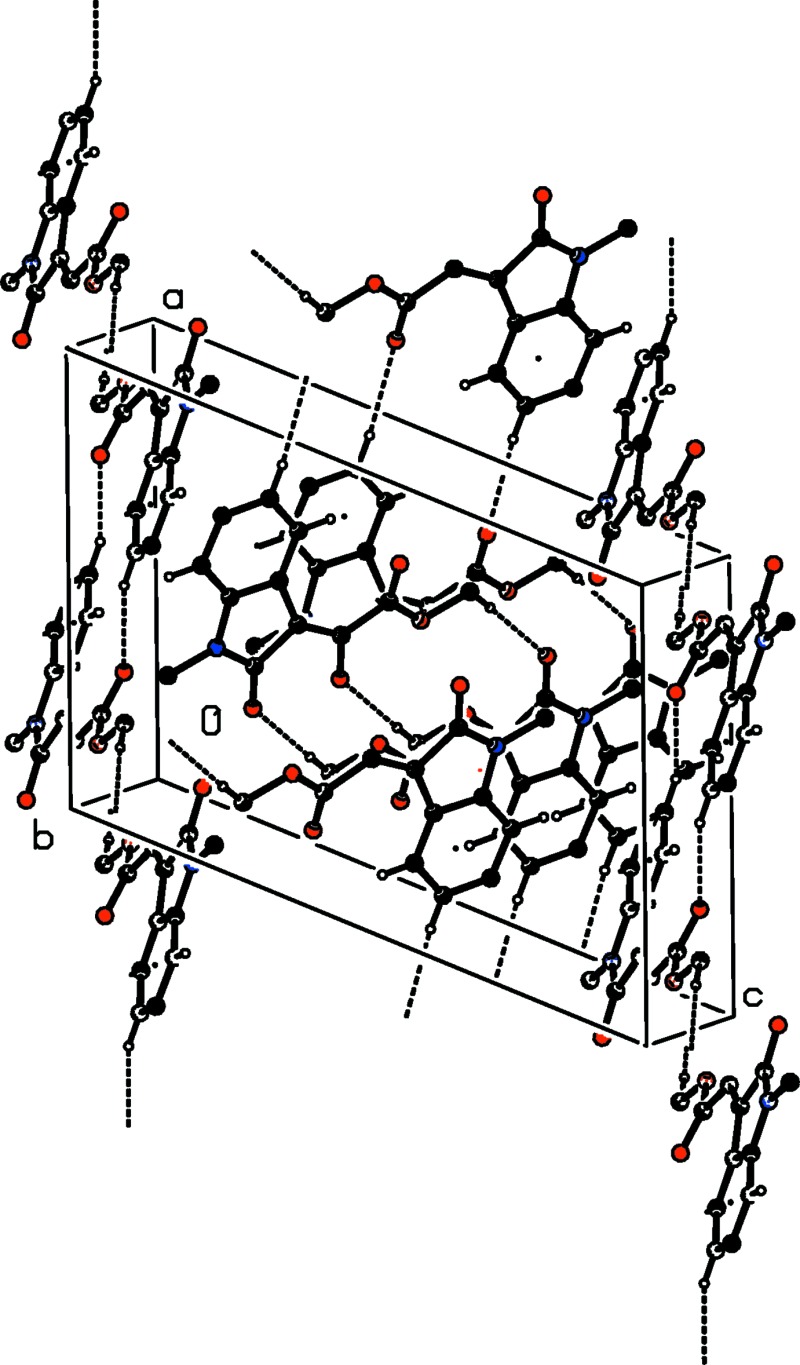
The crystal packing of the title compound viewed along the b axis. The hydrogen bonds are shown as dashed lines (see Table 1 for details; H atoms not involved in hydrogen bonding have been omitted for clarity).

. DOI: 10.1107/S2056989015003217/su5085fig1.tif
The mol­ecular structure of the title compound, with atom labelling. Displacement ellipsoids are drawn at the 30% probability level.

b . DOI: 10.1107/S2056989015003217/su5085fig2.tif
A partial view along the *b* axis of the crystal packing of the title compound. The hydrogen bonds are shown as dashed lines (see Table 1 for details; H atoms not involved in hydrogen bonding have been omitted for clarity).

b . DOI: 10.1107/S2056989015003217/su5085fig3.tif
The crystal packing of the title compound viewed along the *b* axis. The hydrogen bonds are shown as dashed lines (see Table 1 for details; H atoms not involved in hydrogen bonding have been omitted for clarity).

CCDC reference: 1049502

Additional supporting information:  crystallographic information; 3D view; checkCIF report

## Figures and Tables

**Table 1:** Hydrogen-bond geometry (, ) *Cg*1 is the centroid of ring C6C11.

*D*H*A*	*D*H	H*A*	*D* *A*	*D*H*A*
C8H8O2	0.93	2.29	2.988(2)	132
C9H9O2^i^	0.93	2.50	3.387(2)	159
C1H1*A*O3^ii^	0.96	2.57	3.526(2)	175
C11H11*Cg* ^iii^	0.93	2.83	3.558(2)	135

Symmetry codes: (i) 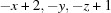; (ii) 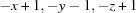; (iii) 
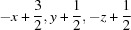
.

## supplementary crystallographic information

### S1. Comment

The indole skeleton is a key component of many biologically active compounds and 3-substituted indole derivatives have been evaluated as precursors of potent anti-inflammatory and analgesic agents (Radwan *et al.*, 2007). Herein, we report on the synthesis and crystal structure of the title compound.

In the title compound (Fig. 1), all bond lengths and angles are normal and comparable with those reported for similar structures (Bhella *et al.*, 2009; Hou & Li, 2011). The five-membered ring (N1/C4-C7) and the six-membered ring (C6-C11) of the the indole group are almost co-planar, with a dihedral angle of 1.76 (8)°.

In the crystal, molecules are linked by pairs of C-H···O hydrogen bonds forming inversion dimers, with an R^2^_2_(16) ring motif (Table 1 and Fig. 2). The dimers are linked by a second pair of C-H···O hydrogen bonds, enclosing R^2^_2_(16) ring motifs, forming ribbons lying parallel to (114); see Table 1 and Fig. 2. The ribbons are linked via C-H···π interactions (Table 1 and Fig. 3) forming a three-dimensional structure.

### S2. Experimental

A mixture of isatin and 1.5 eq of methylbromoacetate were dissolved in DMF with potassium tert-butoxide as catalyst. Th reaction mixture was refluxed at 353 K for 2 h. On completion of the reaction, monitored by thin layer chromatography, the mixture was extracted with ethyl acetate and water. The product was dried and purified by column chromatography using ethyl acetate and hexane (1:9) as an elutent to afford the title compound (yield: 90 %). Colourless block-like crystals were obtained by slow evaporation of a solution in ethyl acetate at room temperature.

### S3. Refinement

All the H atoms were fixed geometrically and allowed to ride on their parent C atoms: C-H = 0.93 - 0.98 Å with *U*_iso_(H) = 1.5*U*_eq_(C) for methyl H atoms and = 1.2*U*_eq_(C) for other H atoms.

### Crystal data

**Table:**

C_12_H_11_NO_3_	*F*(000) = 456
*M**_r_* = 217.22	*D*_x_ = 1.406 Mg m^−^^3^
Monoclinic, *P*2_1_/*n*	Mo *K*α radiation, λ = 0.71073 Å
Hall symbol: -P 2yn	Cell parameters from 1809 reflections
*a* = 11.6814 (7) Å	θ = 2.6–25.0°
*b* = 5.6106 (4) Å	µ = 0.10 mm^−^^1^
*c* = 16.5299 (11) Å	*T* = 293 K
β = 108.713 (2)°	Block, colourless
*V* = 1026.09 (12) Å^3^	0.35 × 0.30 × 0.30 mm
*Z* = 4	

### Data collection

**Table:**

Bruker Kappa APEXII CCD diffractometer	1809 independent reflections
Radiation source: fine-focus sealed tube	1528 reflections with *I* > 2σ(*I*)
Graphite monochromator	*R*_int_ = 0.023
ω and φ scans	θ_max_ = 25.0°, θ_min_ = 2.6°
Absorption correction: multi-scan (*SADABS*; Bruker, 2004)	*h* = −13→13
*T*_min_ = 0.948, *T*_max_ = 0.955	*k* = −6→6
14793 measured reflections	*l* = −19→19

### Refinement

**Table:**

Refinement on *F*^2^	Hydrogen site location: inferred from neighbouring sites
Least-squares matrix: full	H-atom parameters constrained
*R*[*F*^2^ > 2σ(*F*^2^)] = 0.033	*w* = 1/[σ^2^(*F*_o_^2^) + (0.0431*P*)^2^ + 0.3282*P*] where *P* = (*F*_o_^2^ + 2*F*_c_^2^)/3
*wR*(*F*^2^) = 0.093	(Δ/σ)_max_ < 0.001
*S* = 1.05	Δρ_max_ = 0.18 e Å^−^^3^
1809 reflections	Δρ_min_ = −0.13 e Å^−^^3^
148 parameters	Extinction correction: *SHELXL2014* (Sheldrick, 2015), Fc^*^=kFc[1+0.001xFc^2^λ^3^/sin(2θ)]^-1/4^
0 restraints	Extinction coefficient: 0.008 (2)

### Special details

**Table:**

Geometry. All esds (except the esd in the dihedral angle between two l.s. planes) are estimated using the full covariance matrix. The cell esds are taken into account individually in the estimation of esds in distances, angles and torsion angles; correlations between esds in cell parameters are only used when they are defined by crystal symmetry. An approximate (isotropic) treatment of cell esds is used for estimating esds involving l.s. planes.

### Fractional atomic coordinates and isotropic or equivalent isotropic displacement parameters (Å^2^)

**Table:**

	*x*	*y*	*z*	*U*_iso_*/*U*_eq_	
O1	0.65902 (9)	−0.46212 (19)	0.54283 (6)	0.0431 (3)	
O2	0.80169 (10)	−0.1929 (2)	0.54829 (8)	0.0552 (4)	
O3	0.39479 (9)	0.0890 (2)	0.33278 (7)	0.0482 (3)	
N1	0.53182 (11)	0.3529 (2)	0.31063 (8)	0.0373 (3)	
C1	0.74710 (16)	−0.5928 (3)	0.60928 (11)	0.0522 (5)	
H1A	0.7104	−0.7341	0.6227	0.078*	
H1B	0.7760	−0.4949	0.6593	0.078*	
H1C	0.8135	−0.6366	0.5901	0.078*	
C2	0.69963 (13)	−0.2616 (3)	0.51875 (9)	0.0361 (4)	
C3	0.60129 (13)	−0.1379 (3)	0.45343 (9)	0.0365 (4)	
H3	0.5236	−0.1964	0.4439	0.044*	
C4	0.61193 (12)	0.0491 (3)	0.40656 (9)	0.0337 (3)	
C5	0.49776 (13)	0.1589 (3)	0.34670 (9)	0.0354 (3)	
C6	0.65784 (13)	0.3756 (3)	0.33871 (9)	0.0353 (4)	
C7	0.71103 (13)	0.1927 (3)	0.39651 (9)	0.0344 (3)	
C8	0.83571 (14)	0.1806 (3)	0.42921 (10)	0.0418 (4)	
H8	0.8729	0.0592	0.4667	0.050*	
C9	0.90449 (15)	0.3510 (3)	0.40554 (11)	0.0473 (4)	
H9	0.9884	0.3436	0.4272	0.057*	
C10	0.84974 (16)	0.5317 (3)	0.35007 (11)	0.0489 (4)	
H10	0.8975	0.6462	0.3358	0.059*	
C11	0.72544 (15)	0.5465 (3)	0.31528 (10)	0.0442 (4)	
H11	0.6889	0.6676	0.2774	0.053*	
C12	0.44906 (15)	0.5083 (3)	0.24895 (11)	0.0480 (4)	
H12A	0.4669	0.5039	0.1962	0.072*	
H12B	0.4579	0.6684	0.2706	0.072*	
H12C	0.3676	0.4554	0.2393	0.072*	

### Atomic displacement parameters (Å^2^)

**Table:**

	*U*^11^	*U*^22^	*U*^33^	*U*^12^	*U*^13^	*U*^23^
O1	0.0453 (6)	0.0360 (6)	0.0424 (6)	−0.0008 (5)	0.0062 (5)	0.0067 (5)
O2	0.0384 (7)	0.0578 (8)	0.0610 (8)	−0.0034 (6)	0.0042 (5)	0.0181 (6)
O3	0.0353 (6)	0.0460 (7)	0.0569 (7)	−0.0001 (5)	0.0060 (5)	0.0046 (5)
N1	0.0380 (7)	0.0339 (7)	0.0364 (7)	0.0047 (5)	0.0071 (5)	0.0032 (5)
C1	0.0551 (10)	0.0466 (10)	0.0482 (10)	0.0047 (8)	0.0073 (8)	0.0154 (8)
C2	0.0381 (8)	0.0345 (8)	0.0356 (8)	0.0018 (7)	0.0118 (6)	0.0009 (6)
C3	0.0348 (8)	0.0346 (8)	0.0388 (8)	0.0014 (6)	0.0102 (6)	−0.0010 (6)
C4	0.0352 (8)	0.0329 (8)	0.0320 (7)	0.0034 (6)	0.0092 (6)	−0.0037 (6)
C5	0.0371 (8)	0.0331 (8)	0.0342 (8)	0.0022 (6)	0.0087 (6)	−0.0038 (6)
C6	0.0405 (8)	0.0342 (8)	0.0318 (7)	0.0019 (6)	0.0122 (6)	−0.0040 (6)
C7	0.0381 (8)	0.0338 (8)	0.0317 (7)	0.0013 (6)	0.0117 (6)	−0.0034 (6)
C8	0.0371 (8)	0.0461 (9)	0.0407 (8)	0.0022 (7)	0.0105 (7)	0.0007 (7)
C9	0.0384 (9)	0.0563 (11)	0.0479 (9)	−0.0064 (8)	0.0147 (7)	−0.0029 (8)
C10	0.0518 (10)	0.0488 (10)	0.0500 (10)	−0.0118 (8)	0.0219 (8)	−0.0011 (8)
C11	0.0552 (10)	0.0386 (9)	0.0404 (8)	−0.0013 (8)	0.0175 (7)	0.0016 (7)
C12	0.0505 (10)	0.0393 (9)	0.0464 (9)	0.0080 (7)	0.0046 (7)	0.0053 (7)

### Geometric parameters (Å, º)

**Table:**

O1—C2	1.3303 (18)	C4—C5	1.513 (2)
O1—C1	1.4415 (18)	C6—C11	1.374 (2)
O2—C2	1.1980 (18)	C6—C7	1.404 (2)
O3—C5	1.2149 (17)	C7—C8	1.383 (2)
N1—C5	1.3604 (19)	C8—C9	1.384 (2)
N1—C6	1.4001 (19)	C8—H8	0.9300
N1—C12	1.4498 (19)	C9—C10	1.379 (2)
C1—H1A	0.9600	C9—H9	0.9300
C1—H1B	0.9600	C10—C11	1.382 (2)
C1—H1C	0.9600	C10—H10	0.9300
C2—C3	1.474 (2)	C11—H11	0.9300
C3—C4	1.333 (2)	C12—H12A	0.9600
C3—H3	0.9300	C12—H12B	0.9600
C4—C7	1.463 (2)	C12—H12C	0.9600
			
C2—O1—C1	114.99 (12)	C11—C6—C7	122.19 (14)
C5—N1—C6	110.60 (12)	N1—C6—C7	110.34 (13)
C5—N1—C12	124.50 (13)	C8—C7—C6	118.85 (14)
C6—N1—C12	124.84 (13)	C8—C7—C4	134.50 (14)
O1—C1—H1A	109.5	C6—C7—C4	106.64 (12)
O1—C1—H1B	109.5	C7—C8—C9	119.33 (15)
H1A—C1—H1B	109.5	C7—C8—H8	120.3
O1—C1—H1C	109.5	C9—C8—H8	120.3
H1A—C1—H1C	109.5	C10—C9—C8	120.56 (15)
H1B—C1—H1C	109.5	C10—C9—H9	119.7
O2—C2—O1	123.65 (14)	C8—C9—H9	119.7
O2—C2—C3	125.93 (14)	C9—C10—C11	121.47 (15)
O1—C2—C3	110.41 (13)	C9—C10—H10	119.3
C4—C3—C2	126.90 (14)	C11—C10—H10	119.3
C4—C3—H3	116.5	C6—C11—C10	117.57 (15)
C2—C3—H3	116.5	C6—C11—H11	121.2
C3—C4—C7	136.38 (14)	C10—C11—H11	121.2
C3—C4—C5	118.26 (13)	N1—C12—H12A	109.5
C7—C4—C5	105.36 (12)	N1—C12—H12B	109.5
O3—C5—N1	125.89 (14)	H12A—C12—H12B	109.5
O3—C5—C4	127.13 (14)	N1—C12—H12C	109.5
N1—C5—C4	106.98 (12)	H12A—C12—H12C	109.5
C11—C6—N1	127.47 (14)	H12B—C12—H12C	109.5
			
C1—O1—C2—O2	1.0 (2)	C12—N1—C6—C7	−178.31 (13)
C1—O1—C2—C3	−177.63 (13)	C11—C6—C7—C8	−1.7 (2)
O2—C2—C3—C4	11.4 (3)	N1—C6—C7—C8	177.86 (12)
O1—C2—C3—C4	−170.09 (14)	C11—C6—C7—C4	179.26 (13)
C2—C3—C4—C7	2.6 (3)	N1—C6—C7—C4	−1.18 (15)
C2—C3—C4—C5	−176.20 (13)	C3—C4—C7—C8	4.6 (3)
C6—N1—C5—O3	−177.71 (14)	C5—C4—C7—C8	−176.43 (16)
C12—N1—C5—O3	−0.1 (2)	C3—C4—C7—C6	−176.56 (16)
C6—N1—C5—C4	2.20 (15)	C5—C4—C7—C6	2.39 (14)
C12—N1—C5—C4	179.83 (13)	C6—C7—C8—C9	1.2 (2)
C3—C4—C5—O3	−3.7 (2)	C4—C7—C8—C9	179.95 (15)
C7—C4—C5—O3	177.08 (14)	C7—C8—C9—C10	0.1 (2)
C3—C4—C5—N1	176.35 (13)	C8—C9—C10—C11	−1.2 (3)
C7—C4—C5—N1	−2.83 (15)	N1—C6—C11—C10	−178.78 (14)
C5—N1—C6—C11	178.83 (14)	C7—C6—C11—C10	0.7 (2)
C12—N1—C6—C11	1.2 (2)	C9—C10—C11—C6	0.7 (2)
C5—N1—C6—C7	−0.70 (16)		

### Hydrogen-bond geometry (Å, º)

Cg1 is the centroid of ring C6–C11.

**Table:**

*D*—H···*A*	*D*—H	H···*A*	*D*···*A*	*D*—H···*A*
C8—H8···O2	0.93	2.29	2.988 (2)	132
C9—H9···O2^i^	0.93	2.50	3.387 (2)	159
C1—H1*A*···O3^ii^	0.96	2.57	3.526 (2)	175
C11—H11···*Cg*^iii^	0.93	2.83	3.558 (2)	135

Symmetry codes: (i) −*x*+2, −*y*, −*z*+1; (ii) −*x*+1, −*y*−1, −*z*+1; (iii) −*x*+3/2, *y*+1/2, −*z*+1/2.
