# Supplementary figures and images for: Crystal structure of methyl (E)-2-(1-methyl-2-oxoindolin-3-yl­idene)acetate
Source: Acta Crystallogr E Crystallogr Commun. 2015 Feb 21;71(Pt 3):o188–9. doi: 10.1107/S2056989015003217 (PMC4350746; doi:10.1107/S2056989015003217)

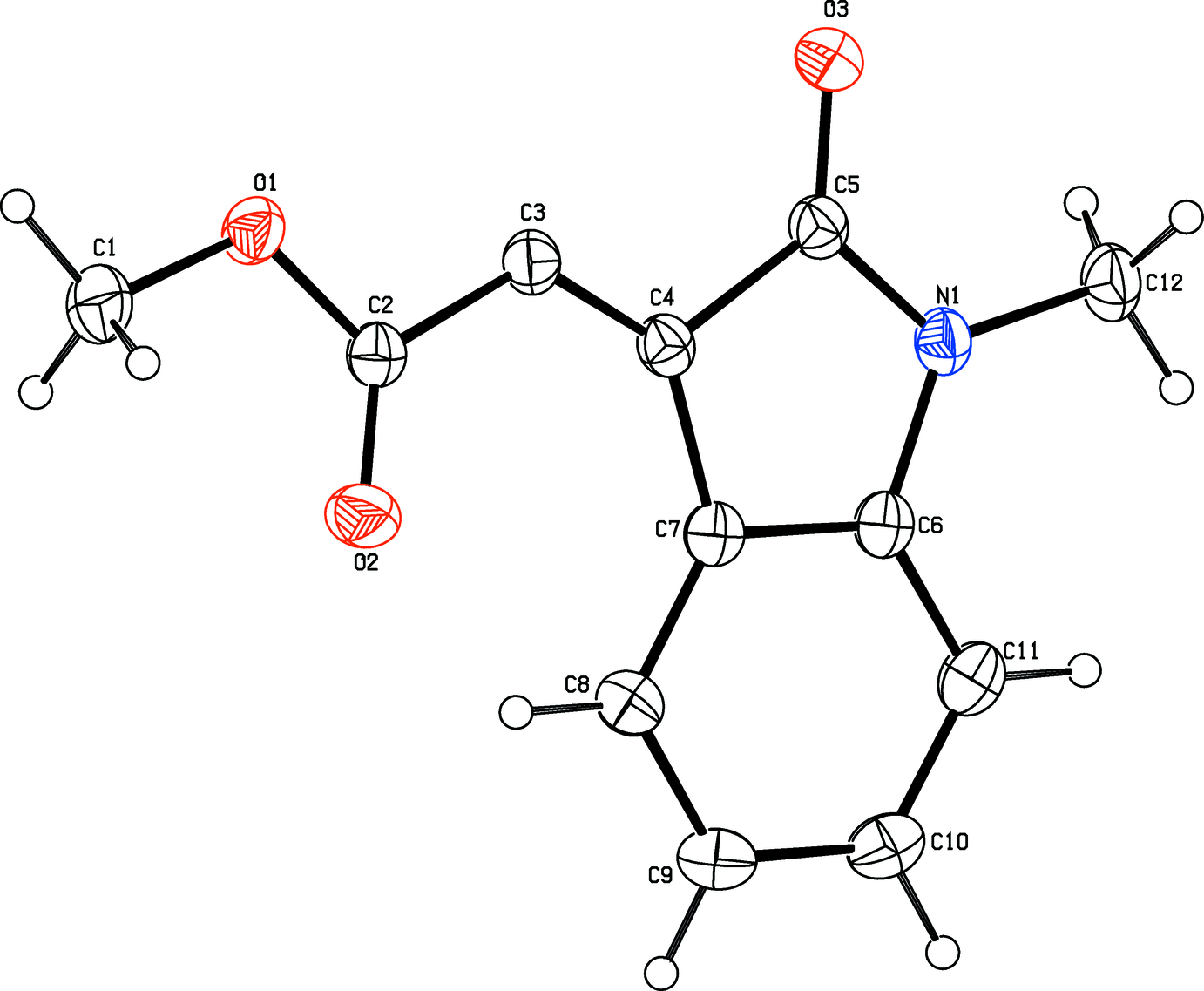

Supplement: Supplementary file 4 [file e-71-0o188-fig1.tif]

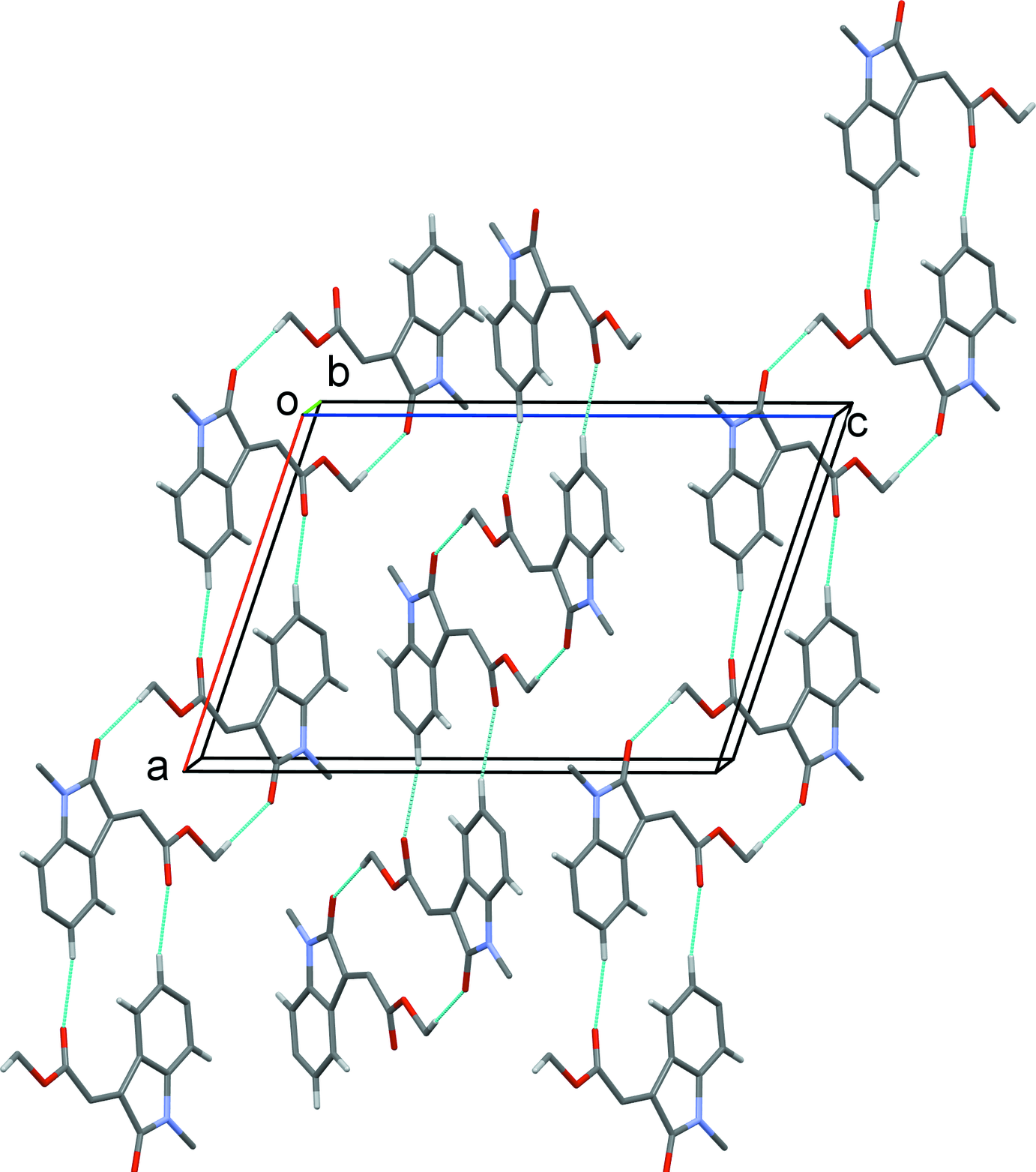

Supplement: Supplementary file 5 [file e-71-0o188-fig2.tif]

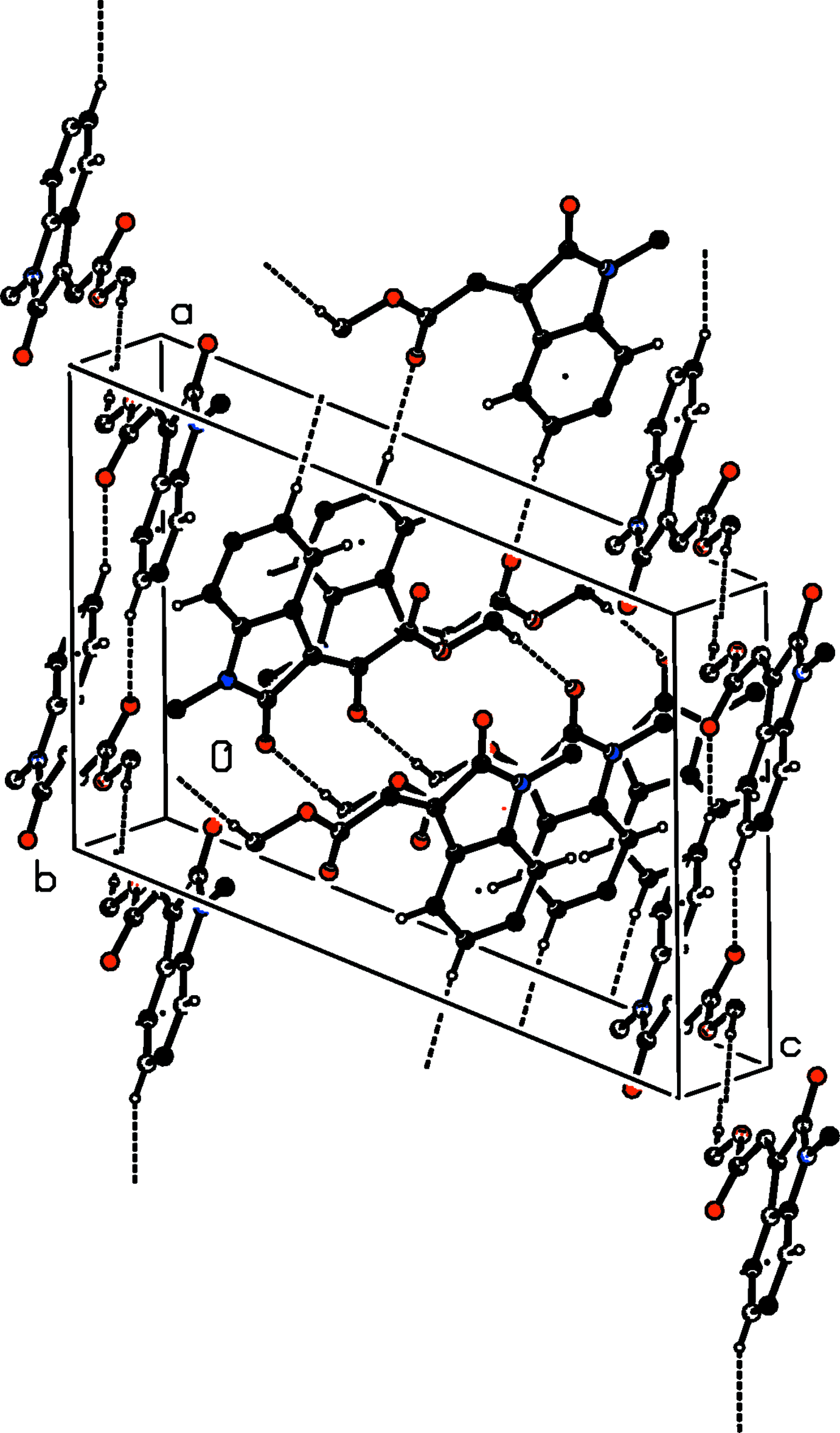

Supplement: Supplementary file 6 [file e-71-0o188-fig3.tif]
